# Supplementary material for: TRPV4 activation triggers protective responses to bacterial lipopolysaccharides in airway epithelial cells
Source: Nat Commun. 2017 Oct 20;8:1059. doi: 10.1038/s41467-017-01201-3 (PMC5651912; doi:10.1038/s41467-017-01201-3)
Supplement: Supplementary file 1 — Supplementary Information [file 41467_2017_1201_MOESM1_ESM.pdf]

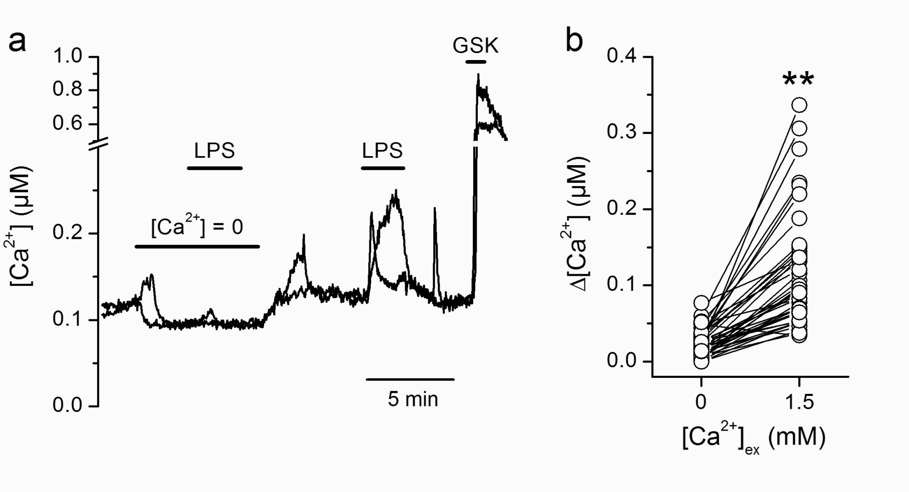

Supplementary Figure 1. LPS-induced Ca^{2+} responses of mTEC require extracellular Ca^{2+}.

(**a**) Representative effects of LPS on the intracellular Ca^{2+} concentration in freshly isolated cells, in the absence and in the presence of extracellular Ca^{2+}. LPS, 20 µg/ml and GSK1016790A, 10 nM. (**b**) Changes in intracellular Ca^{2+} concentration recorded upon stimulation with LPS (20 µg ml^{-1}) in the absence of extracellular Ca^{2+} and after Ca^{2+} restitution. Each data point represents an individual cell in both conditions (n = 47). **, $P < 0.01$, paired *t*-test.

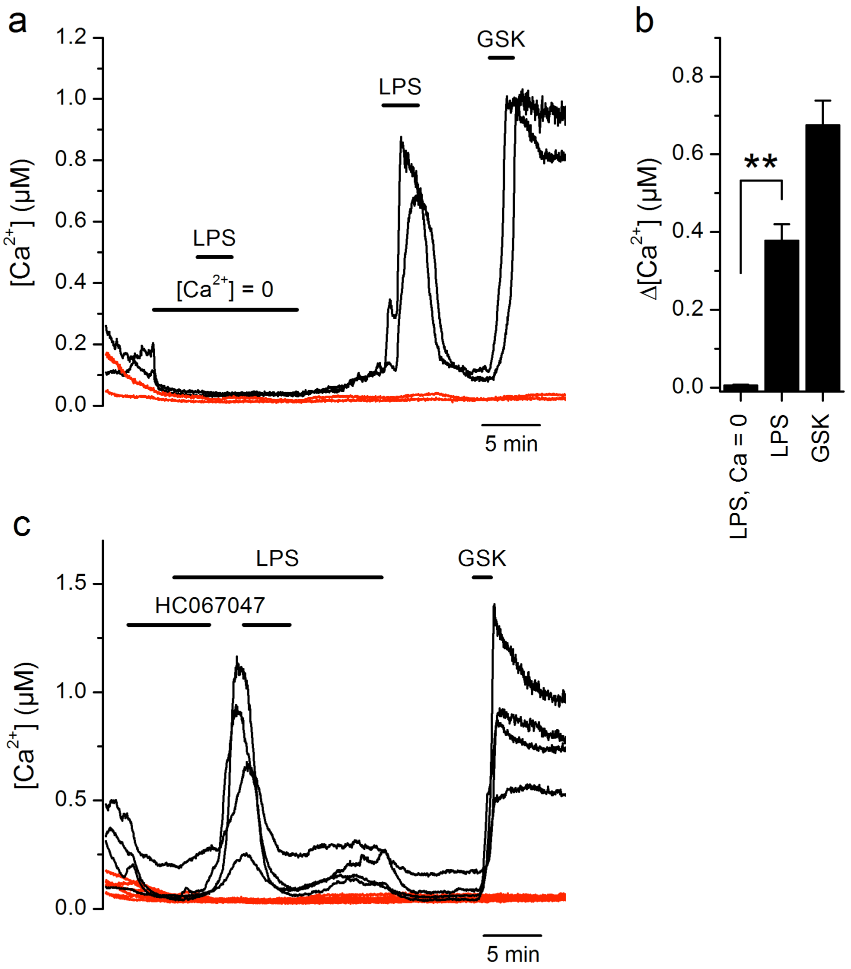

Supplementary Figure 2. LPS induces Ca^{2+} influx through TRPV4 channels in HEK293T cells.

(**a**) Representative traces of intracellular Ca^{2+} signals in TRPV4-transfected HEK293T cells (black traces) showing the lack of LPS-induced responses in absence of extracellular Ca^{2+}. The red traces correspond to non-transfected cells (unresponsive to GSK1016790A). (**b**) Average amplitudes of responses to LPS in the absence extracellular Ca^{2+} and to LPS or GSK1016790A in the presence of extracellular Ca^{2+} (n = 85). **, $P <$ 0.01, Dunn's multiple comparison test. (**c**) Intracellular Ca^{2+} signals in TRPV4-transfected HEK293T cells (black traces) showing the inhibition of the responses elicited by LPS (20 µg ml^{-1}) by the TRPV4 inhibitor HC067047 (10 µM). The red traces were recorded in non-transfected cells (unresponsive to GSK1016790A).

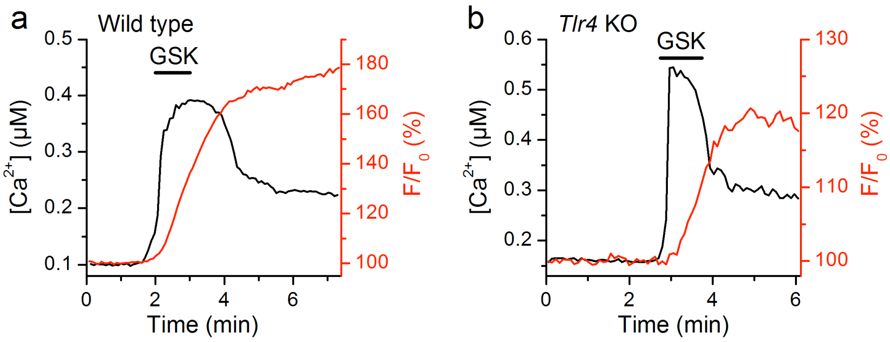

Supplementary Figure 3. Activation of TRPV4 by GSK1016790A induces production of NO in mTEC.

(**a**, **b**) Representative traces of concomitant intracellular Ca^{2+} signals (black solid traces) and normalized fluorescence of NO-sensitive dye DAF-FM (F/F_0, red solid traces) recorded in mTEC harvested from wild type (**a**) and *Tlr4* KO mice (**b**). GSK1016790A, 10 nM.

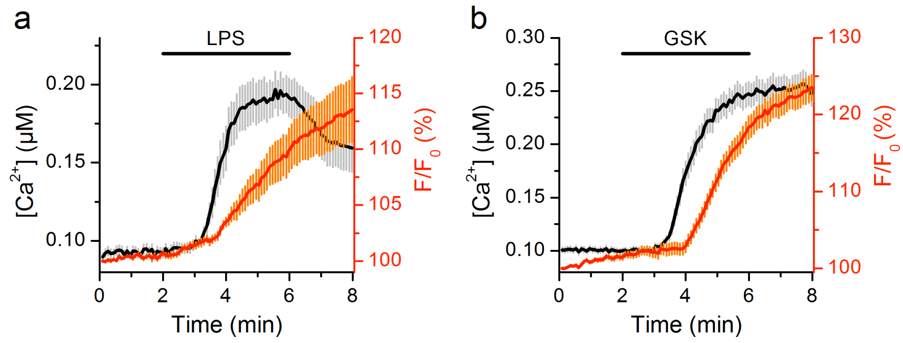

Supplementary Figure 4. Activation of TRPV4 by GSK1016790A induces production of NO in human nasal epithelial cells.

(**a**,**b**) Average intracellular Ca^{2+} signals (black solid traces) and normalized fluorescence of NO-sensitive dye DAF-FM (F/F_0, red solid traces) recorded in freshly isolated human nasal epithelial cells. LPS, 20 µg ml^{-1}; GSK1016790A (GSK) 10 nM.

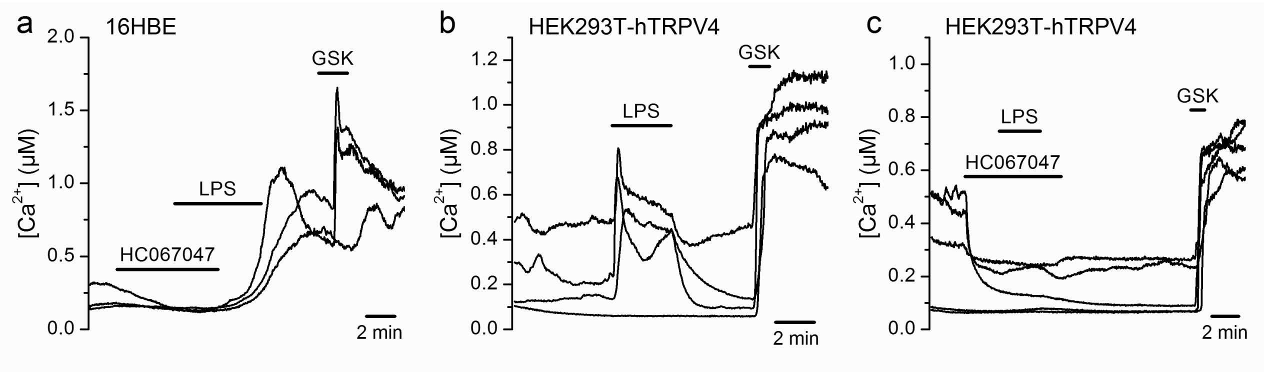

Supplementary Figure 5. LPS-induced activation of human TRPV4 in native and in HEK293T cells.

(**a**) Intracellular Ca^{2+} imaging experiments showing that the TRPV4 inhibitor HC067047 abrogates the responses of 16HBE cells to LPS. (**b**) HEK293T cells transiently transfected with human TRPV4 are stimulated by LPS. (**c**) HC067047 inhibits the responses of hTRPV4-transfected HEK293T cells to LPS. In all panels, LPS 20 µg ml^{-1}; HC067047 10 µM, and GSK1016790A (GSK) 10 nM.

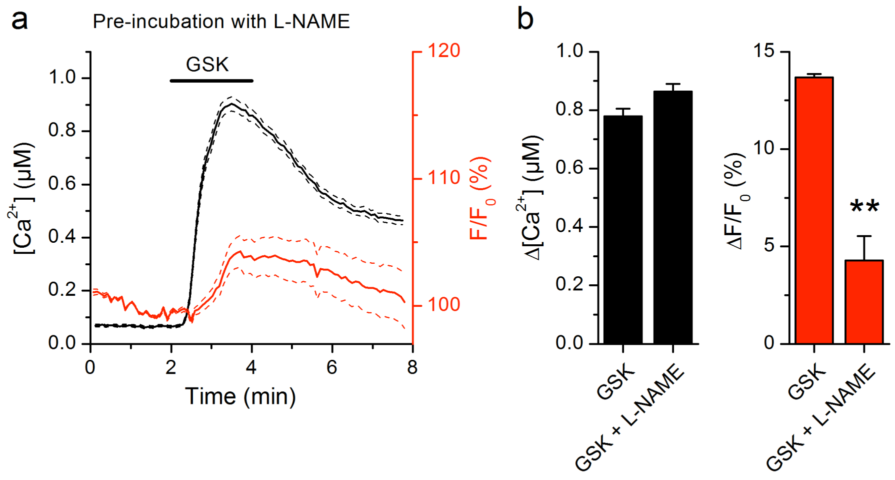

Supplementary Figure 6. LPS-induced activation of TRPV4 elicits enzyme-driven NO production.

(**a**) Average intracellular Ca^{2+} signals (black solid trace) and average normalized fluorescence of NO-sensitive dye DAF-FM (F/F_0, red solid line) in 16HBE cells (n = 144) showing the effects of application of the TRPV4 agonist GSK1016790A (GSK; 10 nM) in cells pre-incubated with the NOS inhibitor L-NAME (1 mM) (n = 249). The thin dashed traces represent the corresponding means ± standard errors. (**b**) Average changes in intracellular Ca^{2+} concentration (left panel) and NO production (right panel) induced by GSK in the absence (n = 144) or presence of L-NAME (n = 249). **, $P < 0.01$, Mann-Whitney *U* test.

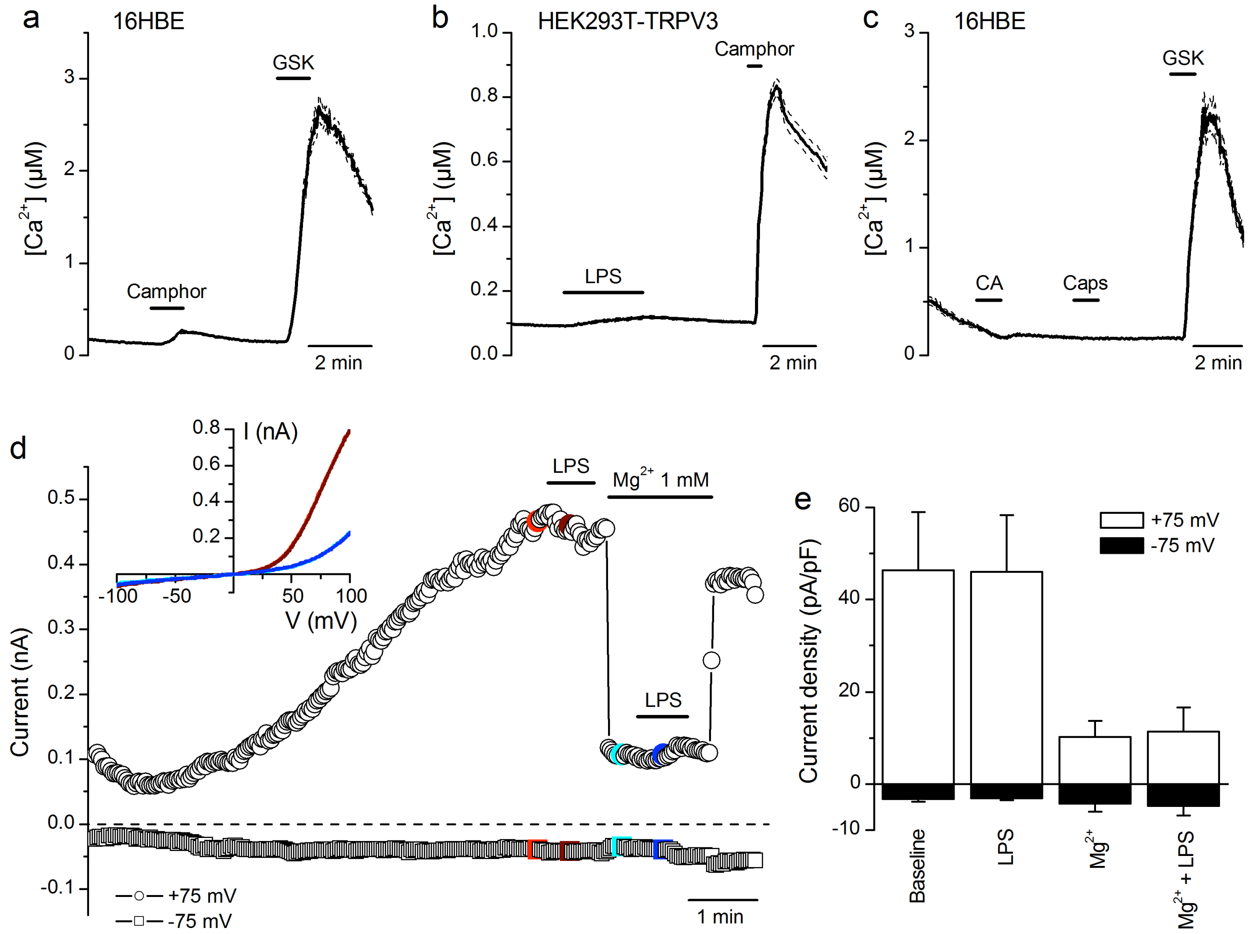

Supplementary Figure 7. Expression of TRPV4 in human bronchial epithelial (16HBE) cells.

(**a**) Average intracellular Ca^{2+} responses of 16HBE cells to the TRPV3 agonist camphor (10 mM) and to the TRPV4 agonist GSK1016790A (GSK; 10 nM) (n = 210). (**b**) Average intracellular Ca^{2+} concentration recorded in HEK293T cells transiently transfected with human TRPV3 (n = 163). These cells do not respond to LPS (20 µg ml^{-1}) but to the TRPV3 agonist camphor (10 mM). (**c**) Average intracellular Ca^{2+} concentration recorded in 16HBE cells (n = 156). These cells do not respond to the TRPA1 agonist cinnamaldehyde (CA, 300 µM) nor to the TRPV1 agonist capsaicin (1 µM), but they respond to GSK1016790A. The thin dashed lines represent mean ± standard error. (**d**) Example of the effects of LPS on the amplitude of currents measured at +75 and −75 mV in a HEK293T cell transfected with mTRPM7. The colored data points correspond to the traces shown in the inset. (**e**) Average current density during baseline and LPS application in the absence or presence of extracellular Mg^{2+} (n = 5).

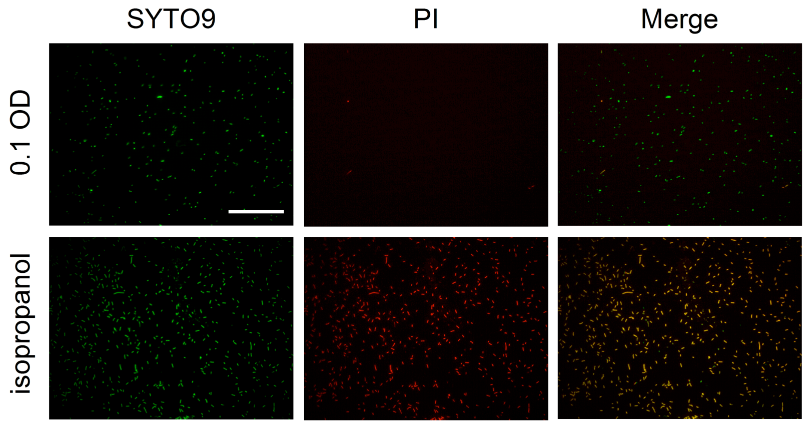

Supplementary Figure 8. Quantification of basal and total cell death in 0.1 OD bacterial cultures.

(**a**) Representative images of *E. coli* cultures stained with SYTO9 (green, all cells) or PI (red, dead cells). Magnification, x40. As a positive control to assess cell death, bacteria were grown until 0.1 OD and later incubated for 15 min in isopropanol. 0.1 OD corresponds to cells imaged immediately after the culture reached 0.1 OD. Scale bar = 50 μm.

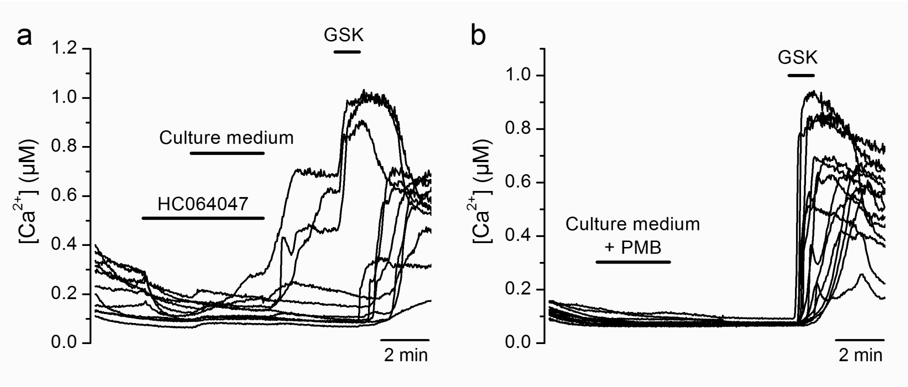

Supplementary Figure 9. 0.1 OD cultures fail to activate TRPV4 in the presence of an TRPV4 antagonist or an LPS scavenger.

(**a**,**b**) Representative traces of intracellular Ca^{2+} concentration in TRPV4-transfected HEK239T cells exposed to the supernatant of 0.1 OD culture of *E. coli* in the presence of HC067047 (10 μM) (**a**) or pre-incubated with polymyxim B (PMB, 300 μg ml^{-1}) (**b**). GSK1016790A, 10 nM.

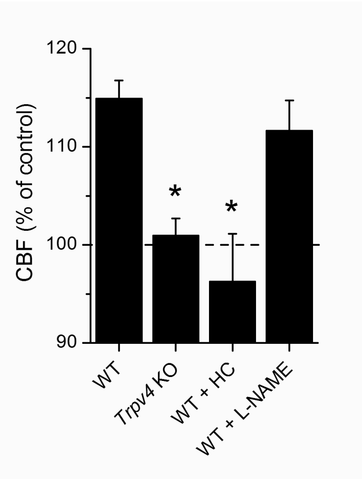

Supplementary Figure 10. LPS increases cilia beat frequency in mTEC.

Relative changes of cilia beat frequency (CBF) recorded upon LPS application to mTEC primary cultures obtained from wild type mice (WT), from *Trpv4* KO mice, or wild type mice in the presence of the TRPV4 inhibitor HC067047 (10 μM) or the presence of the non-selective NOS inhibitor L-NAME (1 mM). *, $P < 0.05$, Dunn's multiple comparison test.

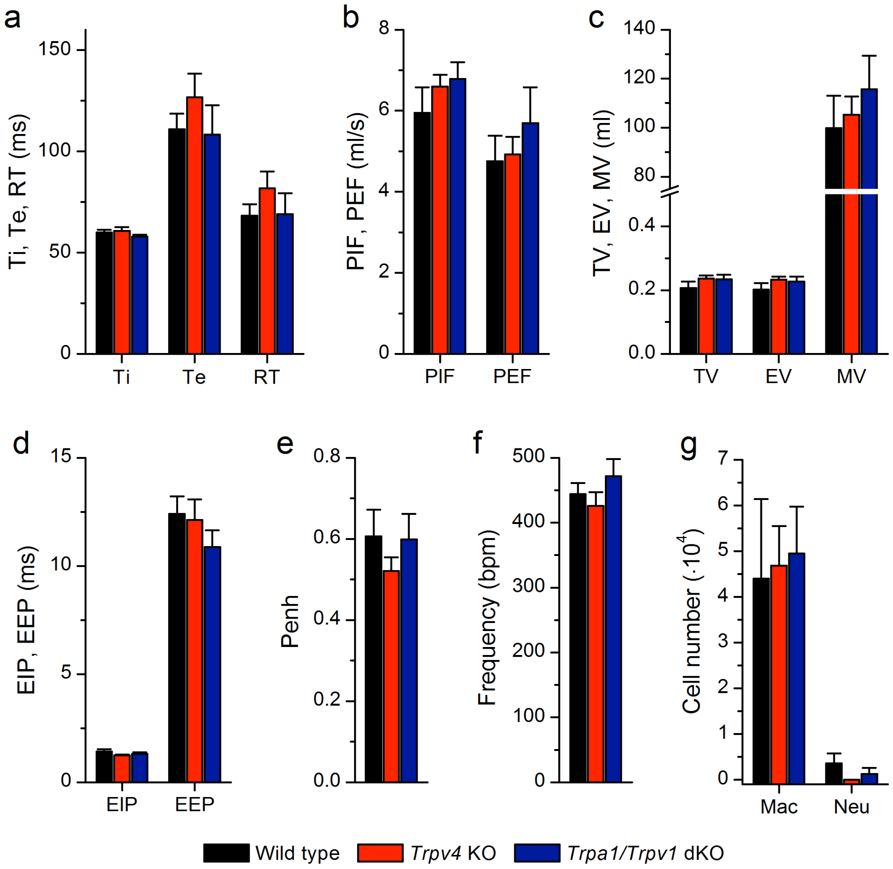

Supplementary Figure 11. Basal ventilatory parameters and airway polymorphonuclear leukocytes in wild type and *Trpv4* KO mice.

(**a-f**) Average basal values of ventilatory parameters in WT (n = 10), *Trpv4* KO (n = 7) and *Trpa1*/*Trpv1* double KO (n = 7) mice. (**g**) Average number of macrophages (Mac) and neutrophils (Neu) in the bronchoalveolar lavage fluid of untreated WT, *Trpv4* KO and *Trpa1*/*Trpv1* double KO animals (n = 5 per genotype).

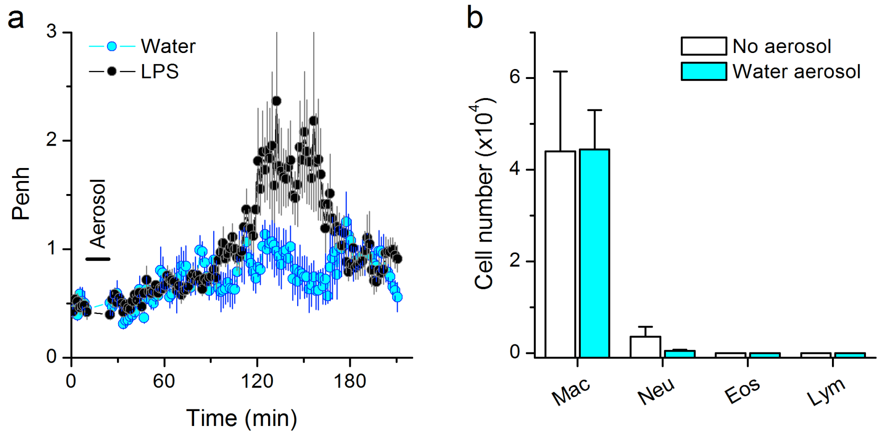

Supplementary Figure 12. Ventilatory parameters and airway polymorphonuclear leukocytes in untreated and LPS-treated wild type mice.

(**a**) Time course of average enhanced pause (Penh) determined in unrestrained whole-body plethysmography experiments performed in wild type mice aerosolized with water during 15 min (n = 5, light blue traces). The data obtained from wild type mice receiving 50 μg ml^{-1} LPS aerosol (same as in Figure 7a) are shown for comparison. Data is represented as mean (symbols) ± standard errors (error bars). (**b**) Average number of leukocytes found in the bronchoalveolar lavage fluid collected from untreated and water-treated mice.

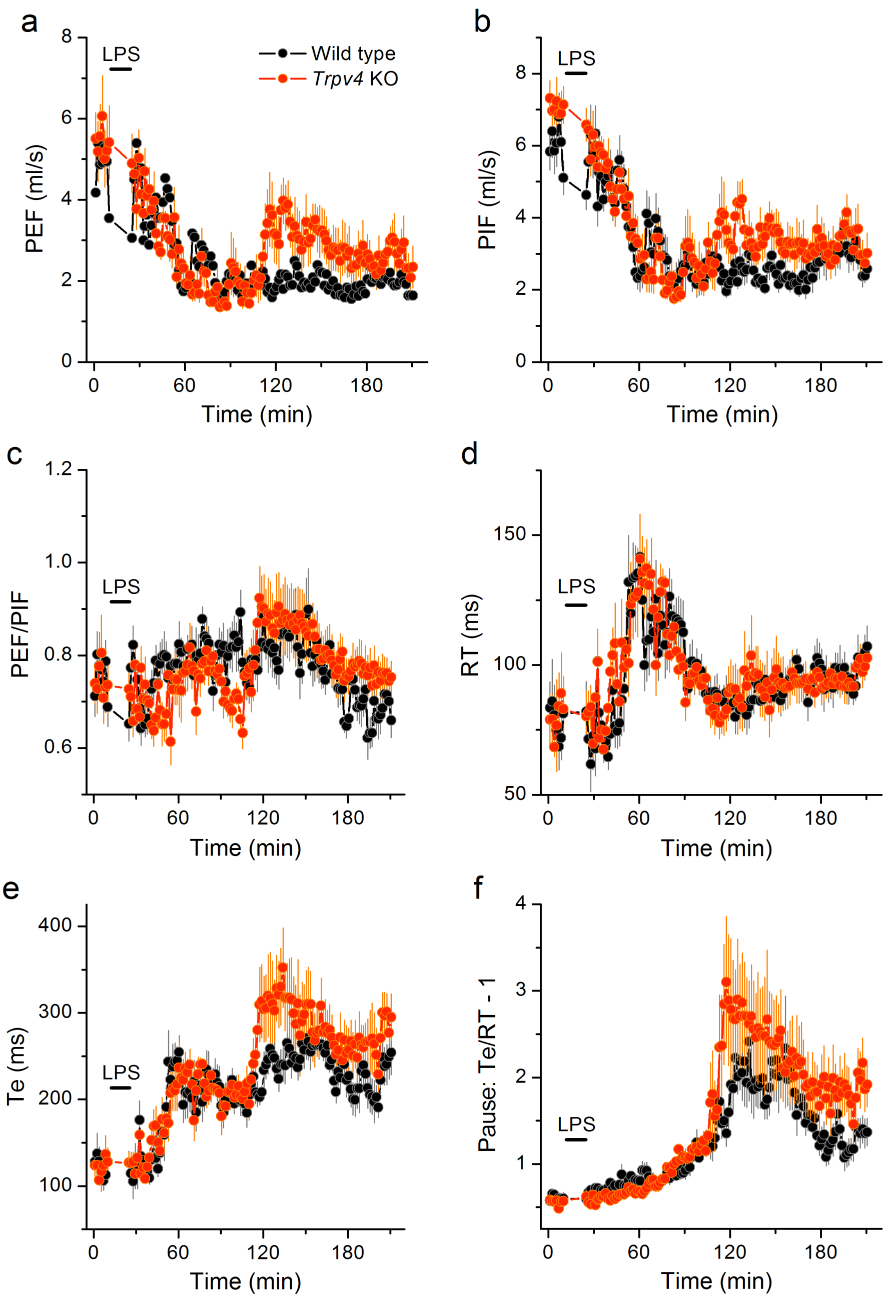

Supplementary Figure 13. Analysis of ventilation parameters defining the enhanced pause.

(**a-f**) Average values of parameters defining the Penh curves shown in Figure 7a. PEF, peak expiratory flow; PIF, peak inspiratory flow; RT, relaxation time and Te, time of expiration. Mice were exposed to aerosols containing 50 µg ml^{-1} LPS for 15 min, as indicated by the horizontal line. The Penh factors PEF/PIF (panel C) and Pause (= Te/RT - 1; panel F) are represented to facilitate the visualization of their individual contribution to Penh. Data is represented as mean (filled symbols) ± standard error (error bars). Note that the change in Te is the major contributor to the difference in Penh response between wild type and *Trpv4* mice. Penh = (PEF/PIF)*Pause = (PEF/PIF)*(Te/RT - 1).

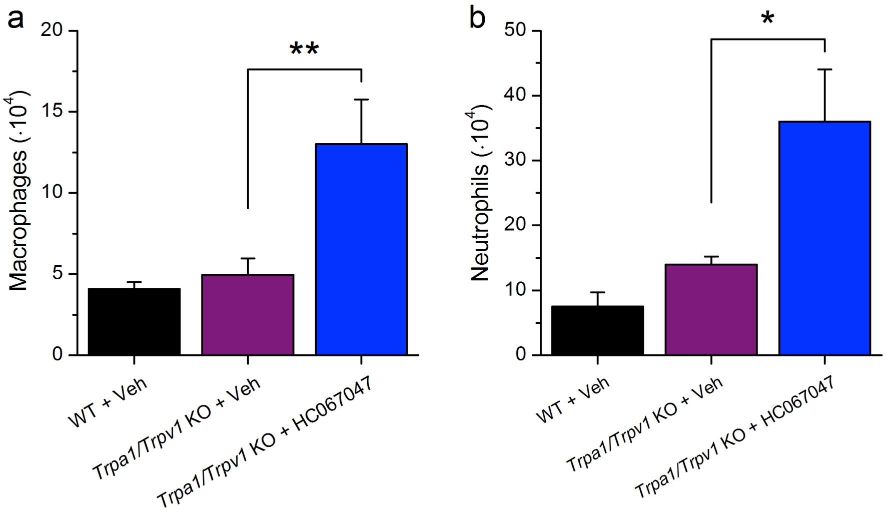

Supplementary Figure 14. Airway cellular inflammatory response to LPS in *Trpa1/Trpv1* KO mice.

(**a, b**) Average number of macrophages (**a**) and neutrophils (**b**) in the bronchoalveolar lavage fluid collected 3 h after LPS exposure (50 µg ml^{-1}, 15 min). WT + Veh, wild type mice pretreated with vehicle (0.8% DMSO), n = 7; *Trpa1/Trpv1* KO + Veh, *Trpa1/Trpv1* KO pretreated with vehicle, n = 6; *Trpa1/Trpv1* + HC067047, *Trpa1/Trpv1* KO pretreated with the TRPV4 inhibitor HC067047 (10 mg kg^{-1} in 0.8% DMSO), n = 6. Veh, vehicle. *, $P < 0.05$; **, $P < 0.01$; two-tailed *t*-test.

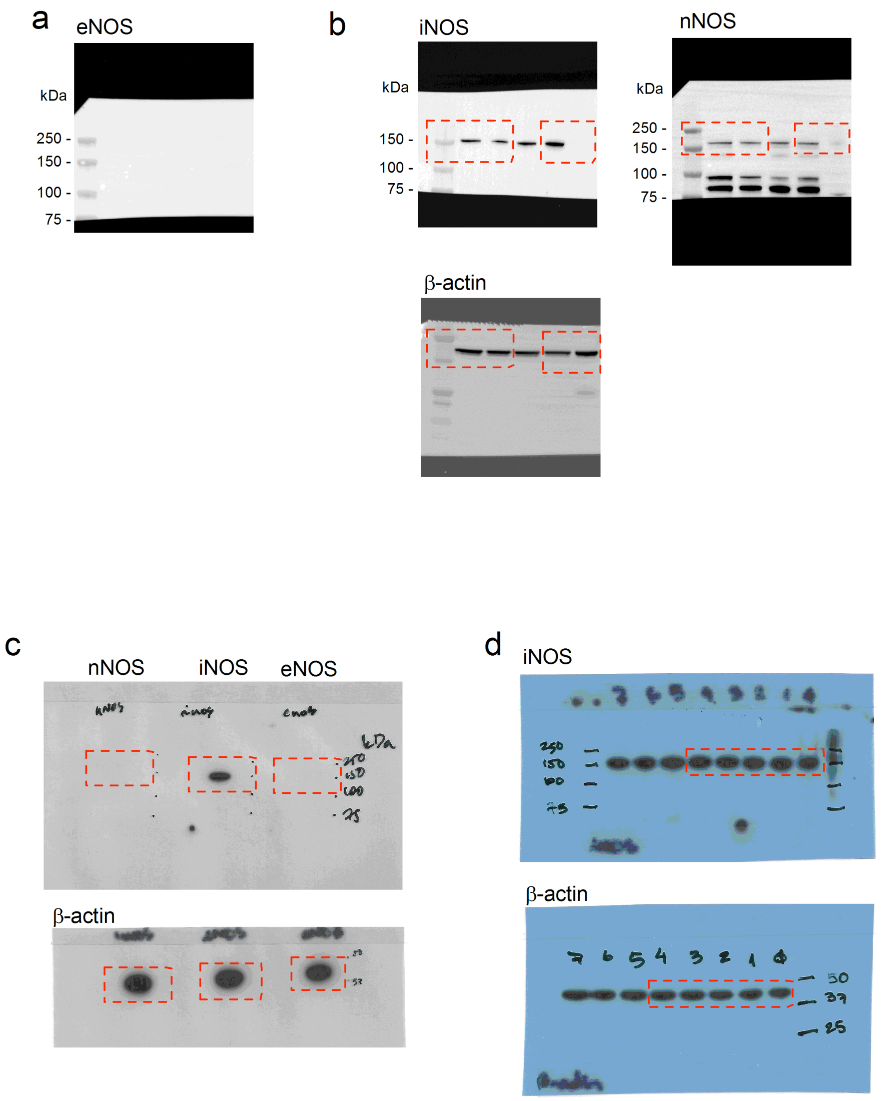

Supplementary Figure 15. Original scans of Western blots. (a) Western blot of mTEC lysates probed for the eNOS. (b-d) Uncropped scans corresponding to images shown in Figure 4g (b), Figure 5a (c) and Figure 5c (d).
